# Phylogeographic patterns of *Calophyllum Braziliense* Camb. *(Calophyllaceae)* based on the psbA-trnH cpDNA locus

**DOI:** 10.1186/1753-6561-5-S7-P17

**Published:** 2011-09-13

**Authors:** Fabiano Salgueiro, Jordana Neri, Marcio Alves-Ferreira, Fabio Scarano

**Affiliations:** 1 Departamento de Botânica, Universidade Federal do Estado do Rio de Janeiro, Rio de Janeiro, Brazil; 2 Programa de Pós-Graduação em Botânica, Instituto de Pesquisas Jardim Botânico do Rio de Janeiro, Rio de Janeiro, Brazil; 3 Departamento de Genética, Universidade Federal do Rio de Janeiro, Rio de Janeiro, Brazil; 4 Departamento de Ecologia, Universidade Federal do Rio de Janeiro, Rio de Janeiro, Brazil

## Background

Past climate changes have severely influenced the current distribution of species and their genetic diversity. Phylogeography is the study of the principles and processes governing the geographic distributions of genealogical lineages [1]. South America has the world's largest area of swamps, floodplains and wetlands in general [2]. Brazil's major wetlands cover 2% of the country's huge territory [3]. However, from a plant ecology standpoint, Brazilian freshwater wetlands are largely unknown and the scarce data available refer mostly to flooded forests of the Amazon [4]. *Calophyllum Braziliense* Camb. (Calophyllaceae), also know as guanandi, jacareúba or landim, is a canopy tree species typical of waterlogged areas from South and Central America. This species occurs in the humid tropical forests of Central America, Amazon Forests, Atlantic Forest (including restingas); and in the riverines forests of the Cerrado biome (brazilian savannah) [5,6]. However, unlike other species typical of flooded areas, *C. Braziliense* shows none of the morphological features common to flood-adapted plants. *Calophyllum Braziliense* is a hermaphroditic tree pollinated by bees. Its seeds are animal (mainly by bats) or water dispersed. The timber has excellent characteristics and is widely used. *Calophyllum. Braziliense* is also used in vegetation restoration programs and its leaves extract presents anti-inflammatory activity [7]. This study examines the phylogeographic patterns of *C. Braziliense* based on the cpDNA intergenic region *psb*A*-trn*H.

## Methods

Twenty four populations of *C. Braziliense* were sampled from Costa Rica (10° 12’N, 83° 47’W) to the Paraná State in Brazil (25° 34’S, 48° 27’W). Samples were collected from about 5-10 adult trees in each population, totaling 192 individuals. Total DNA was extracted from leaves or cambium using the CTAB procedure described by Doyle & Doyle [8]. After a screening for cpDNA amplification and polymorphism in *C. Braziliense*, the *psb*A-*trn*H intergenic regions was selected [9]. Sequences were aligned using CLUSTAL-W implemented in the MEGA 4 software. The cpDNA haplotypes were defined by analyzing the sequences with DNASP 4.01. The genetic diversity indexes were estimated in ARLEQUIN 3.01. The phylogenetic relationships among the haplotypes were estimated using the median-joining algorithm implemented in NETWORK 4.1. An analysis of molecular variance (AMOVA) was performed in ARLEQUIN. A spatial analysis of molecular variance (SAMOVA) was conduced using the SAMOVA 1.0 software. To evaluate the hypothesis of population expansion, neutrality tests were computed in DNASP and ARLEQUIN.

## Results

**Figure 1:**
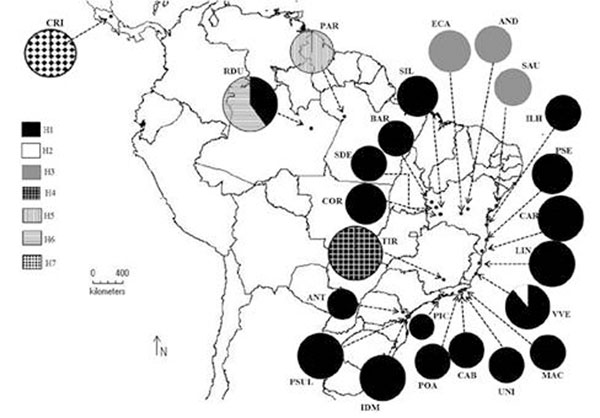
Map showing the geographic distribution of the cpDNA haplotypes in the *Calophyllum Braziliense* populations.

A total of 263 aligned positions were obtained for the *psb*A*-trn*H locus. Twenty-eight variable characters were analyzed resulting in seven cpDNA haplotypes (Figure 1). The haplotype diversity (*h*) for each population ranged from 0.0 to 0.533 and the nucleotide diversity (*π*) from 0.0 to 0.01882. Similar levels of genetic diversity were observed for other tropical species [10,11]. Most of the Atlantic rain forest populations (13/14) are monomorphic and present the same haplotype (H1). Generally, the remaining populations present different private haplotypes. Spatial analysis of molecular variance (SAMOVA) identified seven phylogroups (*k*=7, *Fct* = 0,926), one consisting of 13 monomorphic populations from the Brazilian Atlantic rain forest and more three populations from de Cerrado biome. Most of the others populations constitute different phylogroups. Neutrality tests suggest expansion for the Atlantic rain forest populations. The AMOVA analysis reveals that most of the variation was found between populations (0.8788, *p*<0.00005). A great genetic distance was observed between Central America (Costa Rica) and the others populations, even from the Brazilian Amazon forest. Similar results were observed when *Swietenia macrophylla* (mahogany) populations from Central and South America were compared [10].

## Conclusions

The genetic data obtained here for *C. Braziliense* based on chloroplast DNA diversity indicate a recent expansion for the Atlantic rain forest populations. Our results suggest that the Northeast of Brazil maintained large populations during the last glacial period and that the Southeast and South populations may have undergone a pronounced retraction process, followed by a recolonization process with a strong founder effect. Thus, the recolonization of the South and Southeast region of the Atlantic rain forest probably occurred from these more stable areas in the Northeast.
